# A new knee arthrometer demonstrated to be reliable and accurate to assess anterior tibial translation in comparison with stress radiographs

**DOI:** 10.1007/s00402-022-04679-9

**Published:** 2022-11-07

**Authors:** Giuseppe Milano, Alessandro Colosio, Alessandra Scaini, Marcello Motta, Andrea Raggi, Fabio Zanoni, Stefano Galli, Maristella F. Saccomanno

**Affiliations:** 1 Department of Medical and Surgical Specialties, Radiological Sciences, and Public Health, University of Brescia, Piazzale Spedali Civili 1 c/o Ortopedia e Traumatologia 2, 25123 Brescia, Italy; 2 Department of Biomedical Engineering, FGP Srl, Dossobuono, VR Italy; 3 Department of Bone and Joint Surgery, Spedali Civili, Brescia, Italy

**Keywords:** Knee, Laxity, Anterior cruciate ligament, Anterior tibial translation, Arthrometer

## Abstract

**Introduction:**

For several years, many arthrometers have been developed to assess anterior knee laxity. The aim of our study was to evaluate the validity of a new practical and handy testing device with the hypothesis that the new arthrometer had good validity in terms of reliability and accuracy.

**Methods:**

Lachman test was performed on five fresh frozen cadaveric knees by five examiners. Anterior tibial translation (ATT) was measured with a new arthrometer (BLU-DAT) and on lateral stress radiographs. Data on ATT were obtained under 7 kg (69 N), 9 kg (88 N), and maximum manual traction (MMT). Tests were performed on the same specimens before and after arthroscopic ACL excision. Inter-rater reliability of the BLU-DAT measures was assessed with the intraclass correlation coefficient (ICC) for single and average measurements. The Bland–Altman method was used to estimate agreement between the BLU-DAT and stress radiographs.

**Results:**

ICC values for single measurements were 0.62, 0.54 and 0.58 for 7-kg, 9-kg and MMT assessment, respectively. Overall reliability was good (ICC = 0.63). ICC values for average measurements were 0.89, 0.85 and 0.88 for 7-kg, 9-kg and MMT assessment, respectively. Overall reliability was very good (ICC = 0.90) SEM ranged from 1.4 mm to 1.6 mm for single measurements and was below 1 mm at each testing condition for average measurements. Analysis of agreement between BLU-DAT and radiographic measurements showed a mean difference equal to 0.83 mm ± 2.1 mm (95% CI: 0.55–1.11). Upper LOA was equal to 4.9 mm (95% CI: 5.39–4.41). Lower LOA was equal to − 3.2 mm (95% CI: − 2.71 to − 3.69).

**Conclusion:**

Measurement of anterior knee laxity with the BLU-DAT testing device has a good to very good inter-rater reliability and good agreement with a gold standard such as stress radiographs.

Cadaveric Diagnostic Study, Level of Evidence IV.

## Introduction

The diagnosis of anterior cruciate ligament (ACL) injury is mainly based on clinical examination and confirmed by imaging studies such as magnetic resonance imaging (MRI), which provides valid information on pathoanatomy and has a high correlation with gross pathological findings [1]. However, MRI cannot appraise the functional competence of the ACL.

The Lachman test and the pivot shift test represent the most accurate clinical tests to diagnose an insufficiency of ACL, both in acute and chronic conditions [2–5], albeit its reliability is affected by examiner’s experience and by patient’s compliance as well [4, 6–8].

Quantification of anterior knee laxity has a diagnostic value in case of ACL injury and might help to assess objective outcome of ACL surgery [9–11].

Since the 1980s, several devices named “arthrometers” have been developed to make the knee laxity testing reliable and accurate [12]. However, reliability and diagnostic accuracy of knee arthrometers can be undermined by several factors. Setting-related factors are the knee starting flexion and rotation position [13]. Examiner’s experience, strength, and hand dominance represent the main examiner-related factors [14–16]. Also, patient’s compliance can affect accuracy and reliability of a testing device. Some tools like stress radiographs or radiosterometric analysis (RSA), despite their excellent diagnostic accuracy, are unsuitable for the outpatient setting because of their size or costs [17, 18]. An instrumented laxity testing device should handle all these issues in order to be as accurate and reliable as possible and suitable for every condition of use. Unfortunately, an arthrometer that meet all those features is yet to come. Recently, a new portable testing device (BLU-DAT; FGP srl, Dossobuono, VR, Italy) has been realized for measurement of anterior knee laxity in the clinical setting. The purpose of the present study was to assess validity of this new arthrometer. The hypothesis of the study was that the new arthrometer BLU-DAT has a good validity in terms of reliability and accuracy.

## Methods

The study was designed as a reliability study according to guidelines established by the QAREL checklist [19] approved by our university's institutional review board.

### Study population

Five fresh-frozen lower limbs (femur cut under trochanteric region) from cadavers were used for the present study. Mean donor age was 52.6 ± 13.8 years (range: 31–66 years).

Presence of degenerative changes of the knee joint was assessed on plain radiographs. Diagnostic arthroscopy was performed at the beginning of the procedure to confirm integrity of the ACL. Specimens with evident signs of knee osteoarthritis (joint space narrowing and presence of marginal osteophytes), ACL injuries and radiographic and/or macroscopic signs of previous surgery (hardware, surgical scars) were excluded from the study.

### Description of testing device

**Fig. 1:**
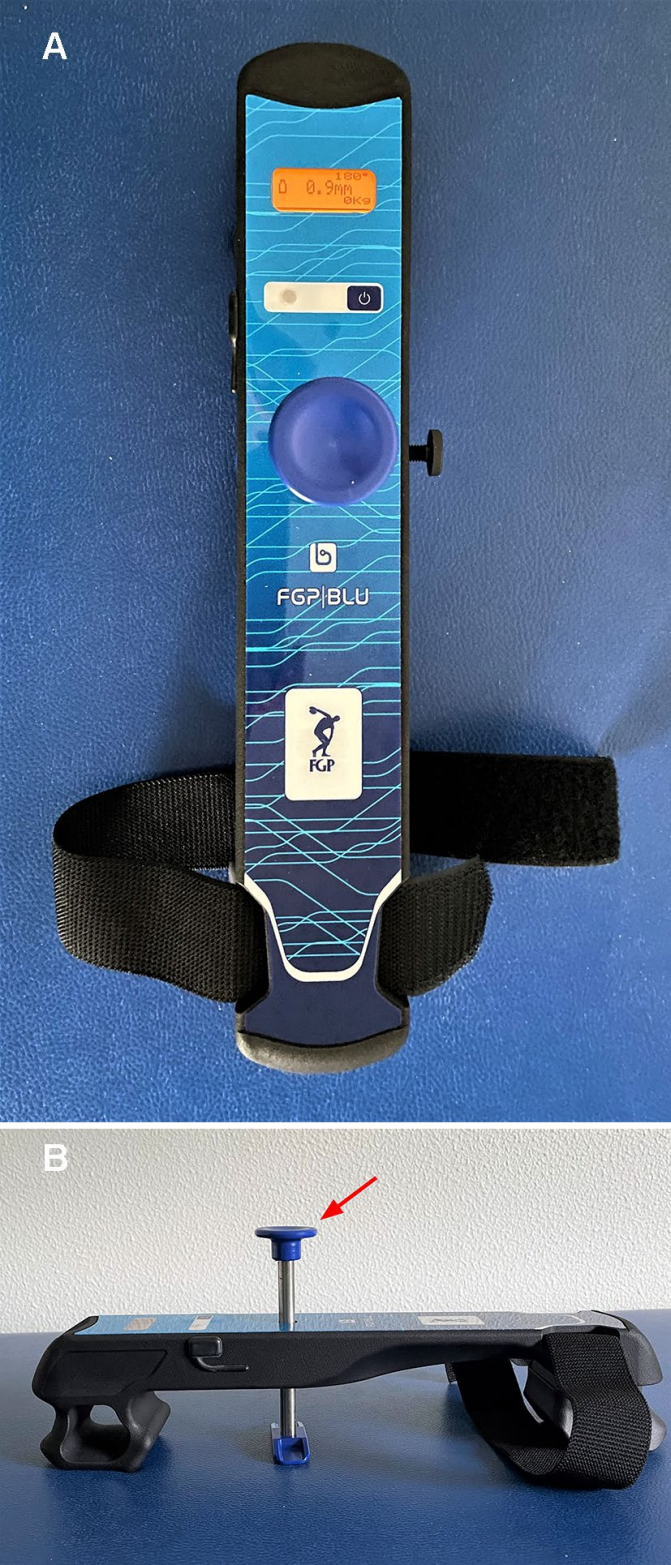
The BLU-DAT laxity testing device (**A**). Displacement on the sagittal plane is measured by a magnetic linear encoder whose mobile part is applied to a sliding rod enveloped in a guiding probe (*arrow*), which is attached to the body of the arthrometer (**B**)

**Fig. 2:**
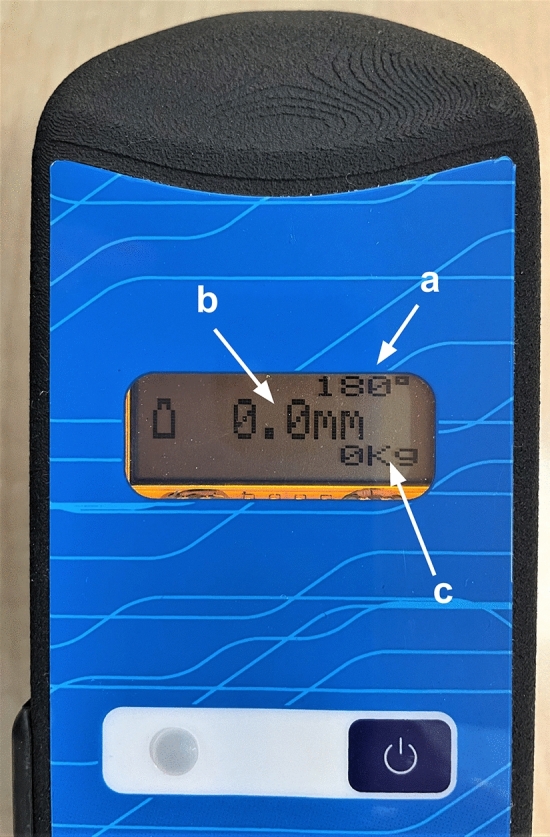
Digital display of the BLU-DAT device. On the display is possible to visualize knee flexion angle (**a**), where 180° corresponds to the plane parallel to the ground; anterior tibial translation expressed in mm (**b**); and the force applied expressed in kilograms (**c**)

The BLU-DAT testing device is designed to measure anterior (or posterior) translation of the tibia respect to the femur. Displacement on the sagittal plane is measured by mean of a magnetic linear encoder whose mobile part is applied to a sliding rod enveloped in a guide (the probe), whereas the feeler is fixed to the arthrometer body (Fig. 1). Measurement of anterior tibia translation relative to the femur is showed on the device display. The device is also equipped by sensors which evaluate the degree of knee flexion during the test, thus allowing to check the proper knee flexion angle according to the clinical testing (i.e., Lachman test and anterior drawer test) (Fig. 2).

**Fig. 3:**
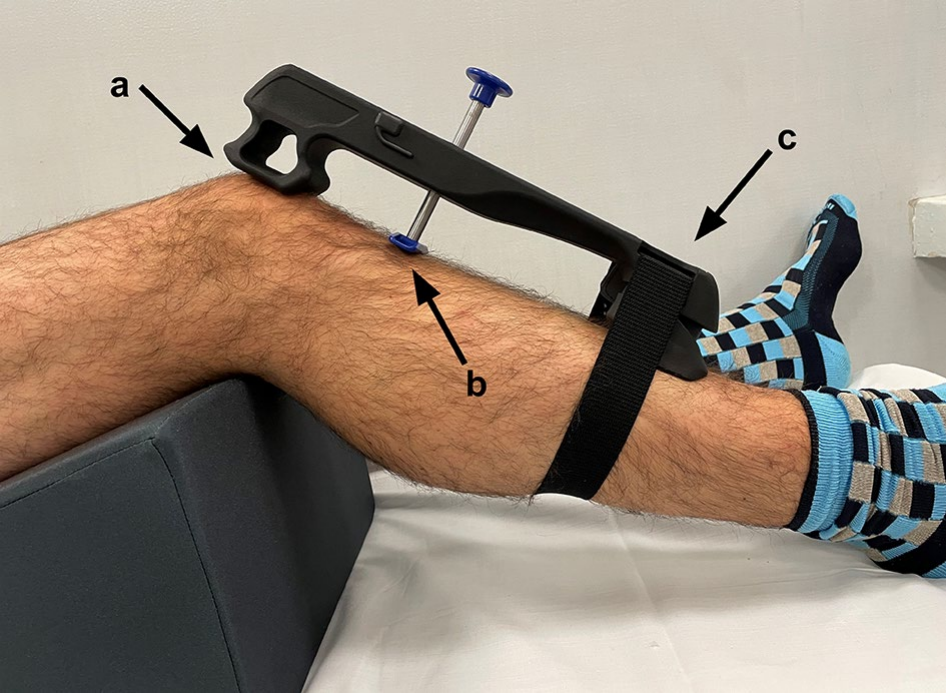
The device in position with the upper support (**a**) positioned on the patella, the probe (**b**) on the tibial tuberosity, and the lower support (**c**) at the level of the distal tibia

**Fig. 4:**
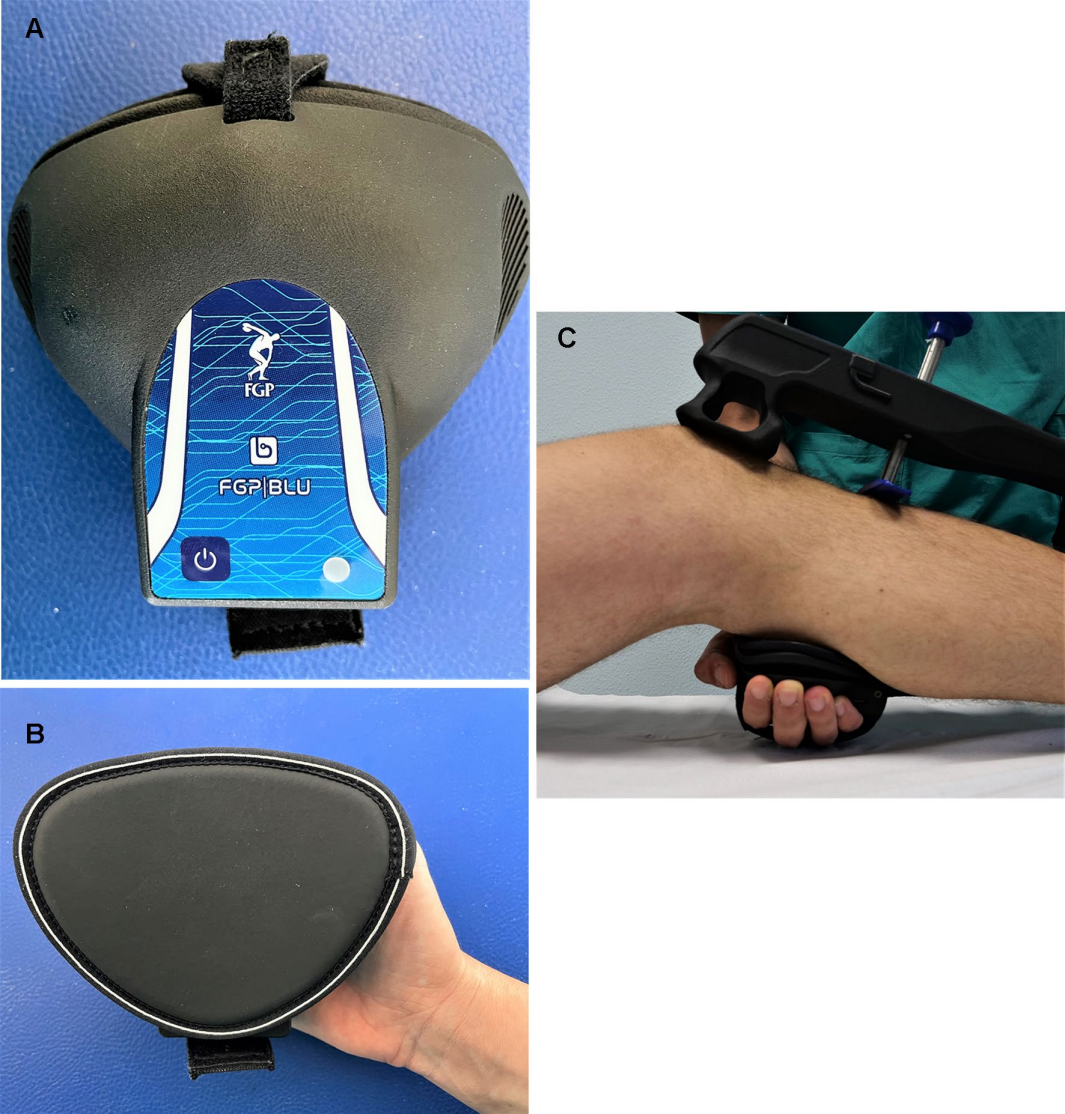
An accessory dynamometer is connected via Bluetooth to the system and allows to quantify the applied force (**A**,** B**). The dynamometer is placed on the examiner’s hand that applies anterior traction to the tibia (**C**)

The arthrometer has two supports: the proximal one should be placed at the level of the patella, whereas the distal one on the distal tibia. The right location of the device is achieved by making the probe falling approximately on the tibial tubercle (Fig. 3). The system can be connected by Bluetooth to an accessory dynamometer that allows to quantify the applied force (Fig. 4). This extension allows to combine displacement data to the force applied while performing the test. The possibility to track the force applied, as well as the knee flexion angle, help to control two important setting conditions that may influence reliability of the test.

### Intervention

Specimens were thawed overnight at room temperature; then lower limbs were mounted over an operating table. A clamp was used to fix the femur, while the foot was fixed to the table with a belt to avoid leg elevation by anterior traction during testing. Knee flexion was set at 30° and in neutral rotation. Three 1-mm titanium beads were positioned into the meta-epiphysis of both femur and tibia from different entry points with the use of a purposed injector through stab incisions. No soft tissues were removed before testing.

Lachman instrumented tests were conducted by five examiners with different skill level (one expert sports medicine surgeon, one sports medicine fellow and three residents with different progress of their residency program—PGY-1, -3 and -5, respectively). Measures of anterior tibial translation (ATT) were acquired under three different loading conditions: 7 kg (69 N), 9 kg (89 N) and maximum manual traction (MMT). An x-ray image intensifier was used while testing. The c-arm of the mobile x-ray unit was placed across the operating table to obtain a lateral view. Preliminary x-ray was obtained to confirm superimposition of the medial and lateral condyles of the distal femur and correct knee flexion angle. Then lateral x-rays were recorded for each test at resting position (no loading) and at the load peak.

Tests were numbered according to the examiner and loading condition and test sequence was randomized by using a random sequence generator (www. random.org). After testing, each specimen underwent an arthroscopic ACL excision, then tests were repeated according to a new random sequence.

Each examiner performed 30 evaluations by testing every specimen 6 times (with and without ACL under three loading conditions). Examiners were blind to test results of other examiners. Overall, 150 tests were carried out.

### Outcome measurements

The primary outcome of the study was the inter-rater reliability of the measures acquired by the five examiners with the BLU-DAT testing device according to different loading conditions. Secondary outcome was the agreement between ATT data obtained with the testing device and those from radiographic images.

Data obtained from manual tests were transmitted via Bluetooth protocol to a PC and acquired by the dedicated BLU-DAT software (FGP). Anterior tibial translation at the force peak under 7 kg, 9 kg and MMT loading conditions were considered. Data were expressed in millimeters (mm).

The output radiographs captured using the “HiRes2-XR” X-Ray machine (Kappa Optronics GmbH, Gleichen, Germany) have been exported with the *dcm* extension. All the collected images were renamed and sorted to match the collection sequence of the BLU-DAT data. Subsequently, the visualization MDICOM software (Kappa Optronics) has been used to convert the renamed files from the *dcm* to *jpg* format to allow resizing of the image when needed. All the images were then imported in the Autocad LT 2020 (Autodesk, San Rafael, CA, USA) CAD software to verify that each set had the correct scale; when correct scale was not confirmed, sets were resized using the Faststone photo resizer software (Faststone Soft) based on the known measure of the diameter of the titanium beads used as reference point.

**Fig. 5:**
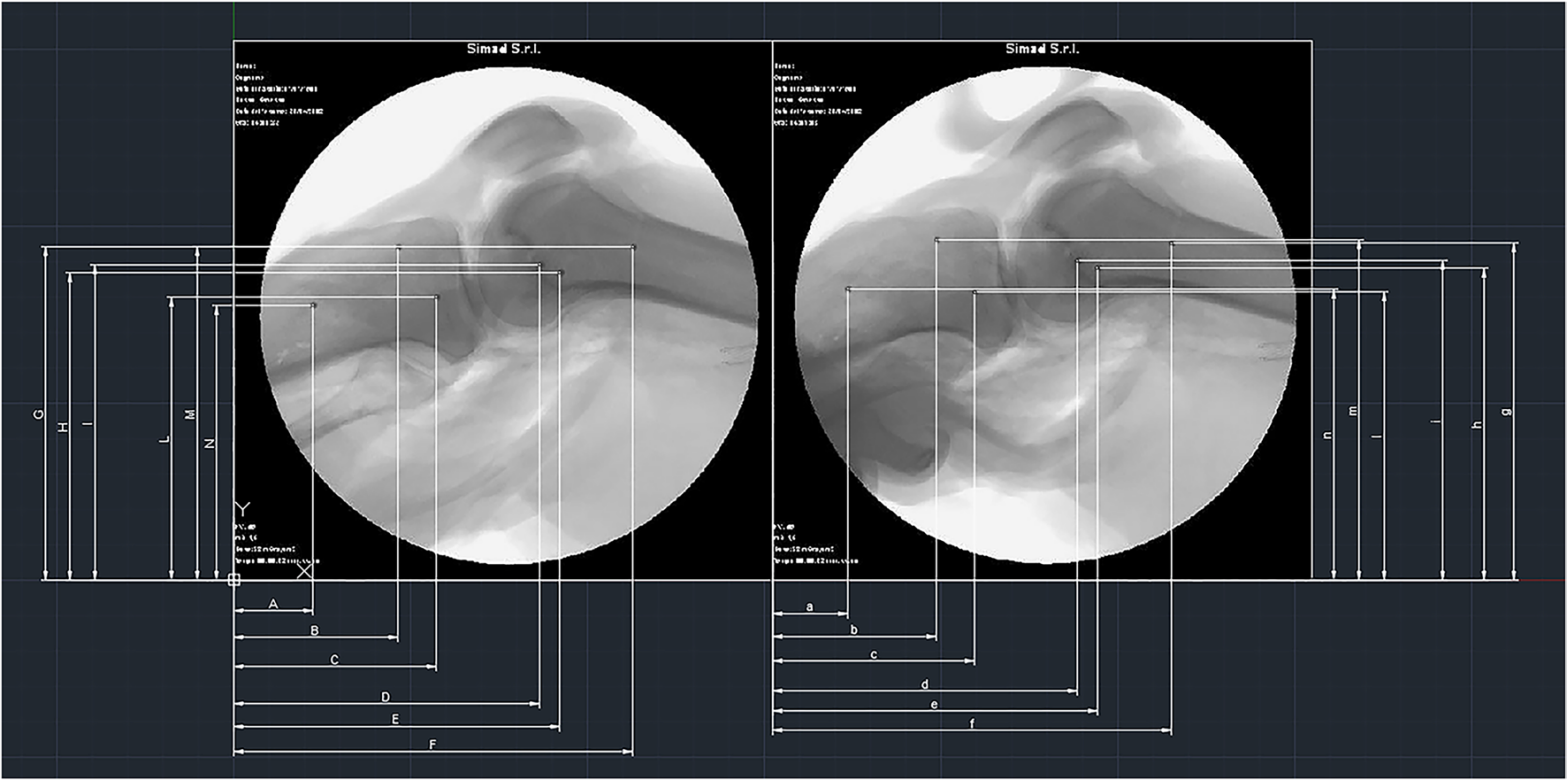
Example of the method used to obtain objective ATT data by stress radiographs, by tracking the differences between the initial position of the tibia (resting phase, RP) and its final position during testing (loading phase, LP). For each of the 6 titanium spheres identified within the radiograph, an X- and Y-coordinate was identified and the X- and Y-components of the cluster of 3 titanium spheres of the tibia and femur were averaged to obtain the exact difference (delta, Δ) of tibial and femoral position between the RP and LP

The approach used to obtain the ATT data consisted of tracking the differences between the initial position of the tibia (resting phase, RP) and its final position during the test (loading phase, LP). The procedure was carried out with Autocad LT 2020 software. For each of the 6 titanium beads identified within the radiograph an X and Y coordinate has been identified and the mean of the X and Y components of the three titanium beads cluster of the tibia and femur was calculated in order to obtain the exact difference (delta, Δ) of the tibial and femoral position between the RP and the LP (Fig. 5).

Then the relative final displacement (RFD) of the tibia respect to the femur was calculated to eliminate any possible error due to femoral movement during the test as follows:

$${\text{RFD}}_{{\text{X}}} = \Delta_{{\text{X}}} {\text{tibia }} - \Delta_{{\text{X}}} {\text{femur}}$$

$${\text{RFD}}_{{\text{Y}}} = \Delta_{{\text{Y}}} {\text{tibia }} - \Delta_{{\text{Y}}} {\text{femur}}$$

The RFD module was obtained by calculating the resultant of the X and Y components of displacement previously obtained:

$${\text{RFD module }} = \, \surd \left( {{\text{RFD}}_{{\text{Y}}} } \right)^{{2}} + \, \left( {{\text{RFD}}_{{\text{X}}} } \right)^{{2}}$$

The differences between the RP and the LP was expressed in mm.

### Data analysis

Data were analyzed with statistical software (IBM SPSS Statistics 25; IBM, Armonk, NY, USA). Normal data distribution was assessed by Kolmogorov–Smirnov test. Descriptive statistics for continuous variables were reported as means and standard deviations. Comparison between raters for ATT measurement at different loading conditions was accomplished by one-way ANOVA. Student’s *t* test was used to compare normal and ACL-deficient knees for ATT measurement at different loading conditions. We analyzed the inter-rater reliability by calculating intraclass correlation coefficient (ICC) using a two-way random effect model and evaluation of absolute agreement. Inter-rater reliability was assessed with the ICC forms for single (2,1) and average (2,k) measurements. Confidence intervals were calculated at 95% confidence level for reliability coefficients. Additionally, from the ICC obtained and the standard deviation of the scores from all subjects, we established the precision by calculating the standard error of measurement (SEM) between observations [20]. ICC values ranged from 0 to 1, with 1 indicating perfect reliability, and they were interpreted as follows: < 0 as absent (complete discordance between observations), 0–0.20 as poor, 0.21–0.40 as fair, 0.41–0.60 as moderate, 0.61–0.80 as good and 0.81–1 as very good [21].

Data obtained from all measurements were pooled and used for analysis of agreement between the two modalities for measurement of anterior tibial translation (testing device and radiographic images). The Bland–Altman method was used to assess agreement between the two measurements for the quantification of ATT [22]. According to this method, difference and mean of the measures obtained from the two measurements were calculated for every laxity test performed [23]. Bland–Altman method was applied by calculating difference of BLU-DAT measure—stress x-ray measure. Agreement was expressed as mean (*d*) and standard deviation (*s*) of the differences. Data were reported in a scatter plot of differences (y-axis) against means (x-axis), where upper and lower limits of agreement (LOA) were considered as *d* ± 2* s.* Ninety-five percent confidence intervals (95% CIs) were calculated for mean difference and estimated LOAs.

### Sample size calculation

Sample size was based on the primary outcome of the study (inter-rater reliability) and established in accordance with estimates provided by Walter et al. [24] for reliability studies using ICCs. A reliability hypothesis at a 5% significance level and a power of 80% (*β* = 0.20) requires a minimum sample of 29 to test inter-rater reliability based on five observers for a criterion coefficient value of 0.5 and a true value of 0.7.

## Results

Comparison between raters for ATT measurement revealed no significant differences between raters at every testing condition (Table 1). Difference in average ATT between normal knees and after ACL cut was significant for every loading condition and at overall evaluation (Table 2).

**Table 1:** Comparison between raters for ATT measurement at different loading conditions

Rater	ATT					
	7 kg	*p*	9 kg	*p*	MMT	*p*
1	2.5 ± 1.2	0.295	3.5 ± 1.5	0.385	4.8 ± 2.5	0.131
2	3.3 ± 2.1		4.1 ± 1.8		5.6 ± 2.3	
3	3.1 ± 1.6		4.3 ± 2		6.2 ± 2.8	
4	3.6 ± 1.8		4.7 ± 2.3		6.2 ± 3.3	
5	2.1 ± 1.6		3.1 ± 2		3.5 ± 1.9	
Overall	2.9 ± 1.7		3.9 ± 2		5.2 ± 2.7

**Table 2:** Comparison between normal and ACL-deficient knees for ATT measurement at different loading conditions

ACL	ATT					
	7 kg	*p*	9 kg	*p*	MMT	*p*
Normal	1.8 ± 0.9	< .0001	2.7 ± 1.7	< .0001	3.7 ± 2.3	< .0001
Cut	4 ± 1.6		5.1 ± 1.4		6.8 ± 2.2	

ICC for single measurements revealed good inter-rater reliability for 7-kg and moderate for 9-kg and MMT assessment. SEM ranged from 1.4 mm to 1.6 mm. Overall, reliability was good. Inter-rater reliability for average measurements was very good for every loading condition and for overall estimate. SEM was below 1 mm for each testing condition (Table 3).

**Table 3:** Reliability analysis for ATT measurement at different loading conditions

Load	Single measurement				Average measurement			
	ICC	95% CI		SEM	ICC	95% CI		SEM
		Lower	Upper			Lower	Upper	
7 kg	0.62	0.35	0.86	1.4	0.89	0.73	0.97	0.7
9 kg	0.54	0.27	0.82	1.6	0.85	0.65	0.96	0.8
MMT	0.58	0.30	0.84	1.5	0.88	0.68	0.96	0.9
Overall	0.63	0.45	0.78	1.4	0.90	0.81	0.95	0.8

**Fig. 6:**
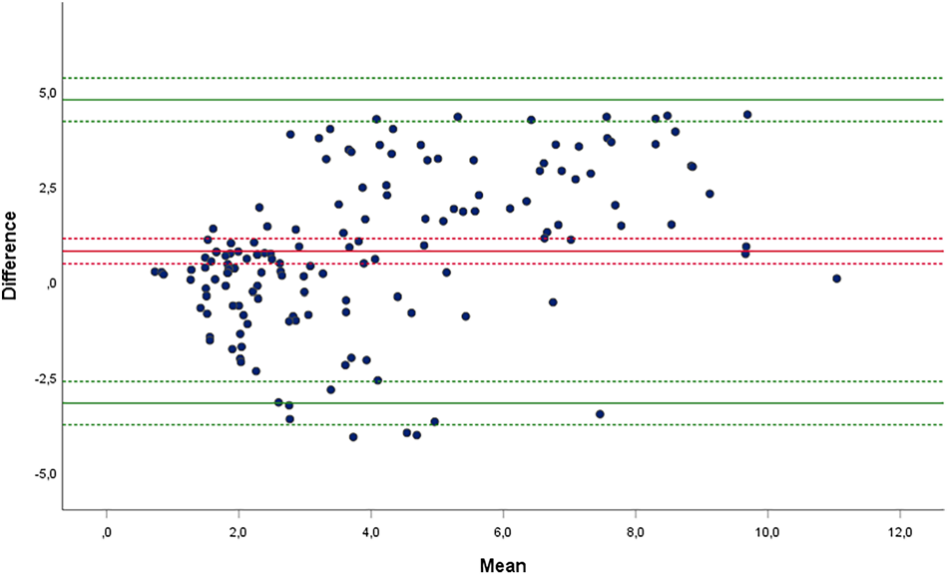
Bland–Altman scatter plot of differences against mean measures of anterior tibial translation (ATT) obtained with BLU-DAT and those obtained on stress radiographs. Difference was calculated as *BLU-DAT measure—stress x-ray measure*. Measures of ATT are expressed in millimeters. The middle (red) line represents the mean difference, and the top and bottom (green) lines represent the limits of agreement (LOA) between the two methods of measurements. Dotted lines represent 95% Cis around mean difference (red dotted line) and around upper and lower LOA (green dotted lines), respectively

Analysis of agreement between BLU-DAT and radiographic measurements showed a mean difference equal to 0.83 mm ± 2.1 mm (95% CI: 0.55–1.11). Upper LOA was equal to 4.9 mm (95% CI: 5.39–4.41). Lower LOA was equal to − 3.2 mm (95% CI: − 2.71 to − 3.69) (Fig. 6).

## Discussion

The most relevant finding of the present study is that reliability of the BLU-DAT testing device in measuring ATT was moderate to good and very good for single and average measurement, respectively. Precision of measurement (SEM) was within 1 mm for average measurements.

In recent decades, several instruments have been developed to quantify ATT. The KT-1000 arthrometer (Medmetric, San Diego, CA, USA) is the hallmark of these tools [12], and most of the new arthrometers have been validated by comparison with the KT-1000 [25–30]. However, reliability of KT-1000 is inconsistently reported in the literature, starting from good and excellent results in older studies [31], to more recent studies that showed lower ICCs, thus definitely downgrading the inter-rater reliability of the KT-1000 [32, 33]. We recall that the KT-1000 has been validated as a diagnostic tool for ACL injuries, hence the output of KT-1000 has been handled as binary or ordinal variable [34]. Conversely, in the present study, reliability of the BLU-DAT testing device was assessed by measuring ATT as continuous variable. Loading conditions were chosen to approximate standard experimental settings (15 lbs, 20 lbs and MMT) to test KT-1000, as reported in previous studies [34–38]. As variability was supposed to be related more to raters than to samples, we attempted to create the worst scenario for assessing reliability by increasing the number of raters (five) with different skills rather than by increasing the number of specimens. What is more, tests could not affect integrity of the specimens, and therefore they were tested several times with no risk of assessment bias due to changes in their mechanical properties. Different raters provided similar mean values for ATT regardless of their skill level and of loading conditions, and this was confirmed at reliability analysis. According to difference in ICC values for single and average measurements observed in the present study, in order to optimize reliability of the instrument, we suggest repeating measurement of ATT three times and calculating average measurement, as recommended for other knee arthrometers [39].

The second aim of this study was to evaluate accuracy of BLU-DAT in measuring ATT by comparison with a gold standard. In the present study, no direct comparison to another established arthrometer (eg. KT1000 or Rolimeter) was accomplished. However, as validity and reliability of other devices are still debated [39], no gold standard has been established to be used for comparison, hence fluoroscopic assessment was considered as the most valid reference standard to estimate real ATT.

Most of previous studies attempted to validate knee laxity testing devices using the correlation coefficient (*r*) as measurement of accuracy. However, correlation studies investigate the relationship between one variable and another, not the differences, and therefore this approach is not recommended as a method for assessing the comparability between methods [40]. The Bland–Altman method used in the present study allows to estimate how much two methods evaluating the same measure differ from each other by calculating the mean difference and the limits of agreement between them [22].

Results of our study confirmed that BLU-DAT has a good agreement with stress radiographs in measuring ATT, with a mean difference between the two measurements being less than one millimeter and LOAs, which represent extreme clinical scenarios, ranging from 4.9 mm to − 3.2 mm.

Bland–Altman method was applied by calculating difference of BLU-DAT measure—stress x-ray measure. As mean difference and 95%CI showed positive results and upper LOA was greater than lower one, we can argue that BLU-DAT has tendency toward overestimation of ATT, albeit within acceptable values. Moreover, by looking at the scatter plot (Fig. 6) we can observe that most of the values of mean difference approximating LOAs are for average ATT exceeding 3 mm, which is considered a threshold for diagnosing an ACL injury [41]. This means that overestimation of anterior knee laxity can occur for greater values of ATT, where diagnosis of ACL injury is already established.

Indeed, some authors evaluated the agreement between knee laxity testing devices and radiological measures [35]. Jorn et al. [42] measured anterior knee laxity in 12 patients after ACL reconstruction using the Stryker laxity tester (Stryker, (Kalamazoo, MI, USA) and RSA simultaneously, with loads of 90 N and 180 N. The mean difference between the two methods was 4.4 mm at 90 N and 8.0 mm at 180 N. The LOA between the two devices were − 1 to + 10 mm at 90 N, and 0 to 16 mm at 180 N, demonstrating a low level of agreement. Shino et al. [41]hypothesized that the lack of agreement between arthrometers and radiological techniques could be due to deformation of the surrounding soft tissues, which affects external device measurements and not the radiological ones.

Although mean difference in ATT between instrumented laxity tests and stress radiographs observed in the present study (less than 1 mm) was much smaller than previously reported [41, 42], some concerns exist about accuracy of BLU-DAT as LOA exceed acceptable values (3 mm-difference). Surely, measurement of ATT by side-to-side difference, as recommended for other arthrometers, might minimize the risk of assessment bias [41]. Unfortunately, due to nature of the present study, performed on single legs, no side-to-side difference could be assessed. Nevertheless, two different laxity conditions were tested for each specimen (intact and torn ACL), which approximated an experimental setting suitable for comparison between injured and contralateral healthy knee. Analysis of ATT before and after ACL cut showed significant difference for every loading condition and at overall evaluation. This means that the device is responsive to change in ATT due to ACL tear, thus confirming its diagnostic accuracy. In support of data obtained in the present study, and for a definitive validation of the instrument, further in vivo studies will certainly be needed to evaluate its reliability and diagnostic accuracy in the clinical setting by side-to-side comparison, as reported in validation studies of other external ATT measurement devices [39].

Finally, we want to focus on manageability of BLU-DAT, given its small size, which makes it an excellent tool for the outpatient setting, overcoming one of the major limitations of other instruments like GNRB, PKTD, and KT-1000 itself. In addition, the optional ability of the instrument to accurately measure force and flexion angle during the examination is crucial to control two of the major setting-related and examiner-related potential confounders.

The present study has some limitations. First, the experiment was conducted on cadaver specimens, making it impossible to test some factors that could have affected the reliability of the new arthrometer in vivo, such as patient compliance and hamstring contraction. Second, accuracy of knee flexion angle was not assessed, nor the exact initial tibial rotation was measured. Finally, no intra-rater reliability was tested.

## Conclusions

Measurement of anterior knee laxity with the BLU-DAT testing device has a good to very good inter-rater reliability and good agreement with a gold standard such as stress radiographs.
